# Preliminary Data Regarding the Potential of Oxytocin to Modulate Aggressive Behaviour in a VPA-Based Animal Model of Autism Spectrum Disorder

**DOI:** 10.3390/ph19020343

**Published:** 2026-02-23

**Authors:** Oana-Georgiana Oprea, Petru Fabian Lungu, Alexandru Ionut Chelaru, Ioana-Miruna Balmus, Roxana Strungaru-Jijie, Gabriel Plavan, Mircea Nicusor Nicoara, Alin Ciobica, Diana Gheban, Stefan Chiriac

**Affiliations:** 1 Doctoral School of Biology, Faculty of Biology, Alexandru Ioan Cuza University of Iasi, No. 20A, Carol I Avenue, 700506 Iasi, Romania; opreageorgiana801@yahoo.ro; 2 Department of Biology, Faculty of Biology, Alexandru Ioan Cuza University of Iasi, No. 20A, Carol I Avenue, 700506 Iasi, Romania; lungufabian123@gmail.com (P.F.L.); gabriel.plavan@uaic.ro (G.P.); mirmag@uaic.ro (M.N.N.); alin.ciobica@uaic.ro (A.C.); 3 Doctoral School of Geosciences, Faculty of Geography and Geology, Alexandru Ioan Cuza University of Iasi, No. 20A, Carol I Avenue, 700506 Iasi, Romania; chelaru.alexandru@yahoo.com; 4 Department of Biology, Faculty of Sciences, “Vasile Alecsandri” University of Bacau, Marasesti Street, 600115 Bacau, Romania; 5 Department of Exact Sciences and Natural Sciences, Institute of Interdisciplinary Research, Alexandru Ioan Cuza University of Iasi, Alexandru Lapusneanu Street, No. 26, 700056 Iasi, Romania; 6 CENEMED Platform for Interdisciplinary Research, “Grigore T. Popa” University of Medicine and Pharmacy, 700115 Iasi, Romania; 7 Research Center Advanced Materials and Technologies (RAMTECH), Department of Exact and Natural Sciences, Institute of Interdisciplinary Research, Alexandru Ioan Cuza University of Iasi, No. 20A, Carol I Avenue, 700506 Iasi, Romania; roxana.jijie@uaic.ro; 8 “Ioan Haulica” Institute, Apollonia University, 700511 Iasi, Romania; 9 Biomedical Research Group, “Olga Necrasov” Center, Romanian Academy, Iasi Branch, Teodor Codrescu 2, 700481 Iasi, Romania; 10 Faculty of Medicine and Biomedical Sciences, Apollonia University of Iasi, Pacurari Street 11, 700511 Iasi, Romania; 11 Department of Gastroenterology, Faculty of Medicine, “Grigore T. Popa” University of Medicine and Pharmacy, 700115 Iasi, Romania; stefannchiriac@yahoo.com

**Keywords:** autism spectrum disorder, oxytocin, risperidone, aggressive behaviour, valproic acid, zebrafish

## Abstract

**Background/Objectives**: Aggressive behaviour is commonly associated with neurodevelopmental disorders, such as autism spectrum disorder (ASD), and could be understood as a response to daily stress routines, which negatively impacts patients’ quality of life. Oxytocin (OT), a neuropeptide involved in social bonding and socio-affective regulation, has emerged as a promising candidate to enrich, rather than replace, current pharmacological approaches in managing ASD-associated aggressive behaviour. In this study, we examined the potential of OT to modulate aggressive behaviour frequency in a VPA-based animal model of ASD. **Methods**: Sixty adult zebrafish (1:1 sex ratio) were divided into six groups (*n* = 10/group) and received the following treatment for 7 consecutive days: CTR—control (no treatment); VPA (28.8 mg/L valproic acid); OT (33.2 ng/mL oxytocin); RIS (170 μg/L risperidone); VPA + OT (28.8 mg/L valproic acid and 33.2 ng/mL oxytocin); and VPA + RIS (28.8 mg/L valproic acid and 170 μg/L risperidone). The locomotor performance, and socio-affective and aggressive behaviours, were measured in the Novel Tank and Mirror Biting tests at the end of the treatments. **Results**: We observed that the VPA treatment led to locomotion and socio-affective impairments, as well as aggressive behaviour. Also, we found that OT and RIS had comparable potential to modulate the frequency of aggressive and anxiety-like behaviours. **Conclusions**: Our preliminary data showed that OT has the potential to modulate the frequency of anxiety-like and aggressive behaviours, similarly to the atypical antipsychotic, RIS, in our VPA zebrafish model. However, further studies are needed to investigate the mechanisms of action and their potential synergistic effects.

## 1. Introduction

Aggressive behaviour could be defined as any deliberate act of inflicting physical or psychological damage to individuals or property [1]. While DSM-5 generically classifies aggressive behaviour as a disorder of impulse control and conduct [2], it could be associated with neurodevelopmental disorders, such as autism spectrum disorder (ASD) [1], implying an altered response to daily stress and decreasing patients’ quality of life. Despite not being a core symptom of ASD, aggressive behaviour could play an important role in the efforts to manage ASD patients, while its pharmacological approaches are currently scarce, with only risperidone (RIS) and aripiprazole being approved by the FDA [3]. Studies have reported that dopamine agonists efficiently improved aggressive behaviour, impulsivity, and stereotypic behaviour [3,4]. The positive effects of other antipsychotics, such as haloperidol, lurasidone, and naltrexone, or serotonin reuptake inhibitors (SSRIs), such as fluoxetine and fluvoxamine, were also described, also used for reducing the symptoms, but not for addressing the underlying causes [3,5].

Oxytocin (OT) is an endogenous polypeptide produced in the hypothalamus and released in the blood circulation by the posterior pituitary gland [6,7]. OT is clinically used for inducing labour, post-partum haemorrhage, lactation, and improving migraine, sexual dysfunctions, and drug addictions [6,8], but also as a therapy adjuvant in neuropsychiatric disorders, such as ASD and schizophrenia [6,7]. In this context, the analgesic effect of OT was reported to contribute to managing pain, anxiety, depressive mood, and stress response in ASD and other psychiatric disorders [6,7,9]. Various animal model studies have described the neurobiological effects of endogenous oxytocin on improving social impairments and associated behavioural manifestations [10,11,12].

Risperidone (RIS) is an atypical antipsychotic antagonising D2 dopaminergic and 5-HT2A serotonin receptors to improve social interactions and reduce aggressive behaviour and irritability [3,5,13]. Despite being widely used to manage ASD patients’ aggressive behaviour, several secondary effects of RIS were reported, mainly due to also being an antagonist of histaminergic H1 receptors, leading to reduced dopaminergic activity, sedation, and increased body weight [13,14].

Due to the ability to perform complex behavioural tasks and increased similarity of brain morphology, neural and signalling pathways with mammalian species, and zebrafish (*Danio rerio*), are widely used in neurobiological research [15,16,17]. In this way, animal models, such as zebrafish, could contribute to the understanding of the neurobiology of ASD, including the pharmacological modulation of ASD-related socio-affective behaviour, such as social withdrawal, social anxiety, and aggressive or repetitive behaviours, which are the result of highly conserved cognitive pathways [16,18,19].

In this context, this study aimed to evaluate and describe the potential of OT to modulate ASD-associated behaviours, especially aggressive behaviour, in a VPA-based adult zebrafish model, as well as to compare the modulatory potential of OT with that of an atypical antipsychotic widely used to treat aggressive behaviour in ASD patients (i.e., RIS).

## 2. Results

### 2.1. The Effect of OT on Exploratory Behaviour

**Figure 1:**
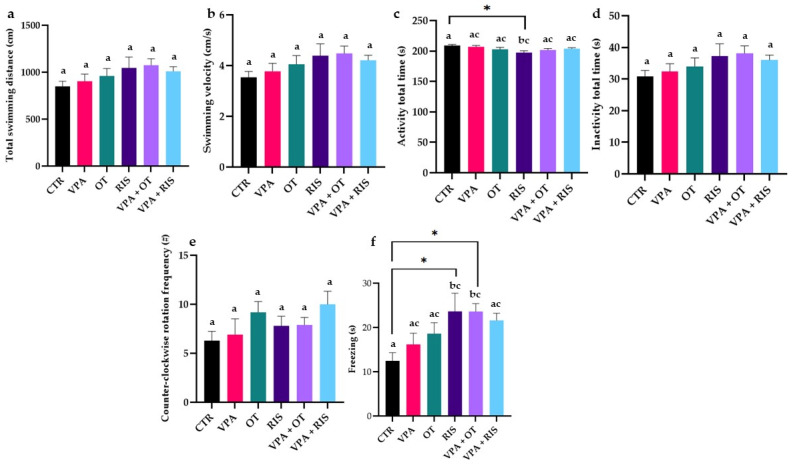
Locomotor performance, as evaluated using NTT: (**a**) Total swimming distance (cm); (**b**) swimming velocity (cm/s); (**c**) activity total time (s); (**d**) inactivity total time (s); (**e**) freezing behaviour duration (s); and (**f**) counter-clockwise rotation frequency (#). The results are expressed as mean ± SEM (CTR—control; VPA—valproic acid exposure; OT—oxytocin; RIS—risperidone; VPA + OT—valproic acid and oxytocin; and VPA + RIS—valproic acid and risperidone); * *p* < 0.05 in two-way ANOVA test; a–c in Tukey’s HSD test.

The total swimming distance, along with the swimming velocity, visibly increased in the VPA-treated groups, as compared to healthy individuals. Total activity time decreased, as compared to the CTR group, especially in the RIS group (*p* < 0.05 in two-way ANOVA test). No significant differences were observed for the inactivity time and counter-clockwise rotation frequency in all experimental groups (Figure 1d,e). On the other hand, freezing time was visibly increased in all groups, as compared to the CTR group (Figure 1f). Significantly higher freezing time was recorded in the RIS (*p* < 0.05 in a two-way ANOVA test) and VPA + OT (*p* < 0.05 in a two-way ANOVA test) groups, as compared to the CTR group, suggesting increased anxiety-like states (Figure 1f).

**Figure 2:**
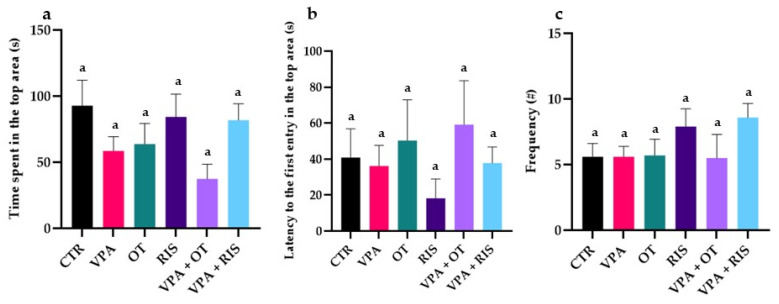
Specific behavioural parameters evaluated using NTT: (**a**) Time spent in the top area (s); (**b**) latency to the first entry in the top area (s); and (**c**) zone alternation frequency (#). The results are expressed as mean ± SEM (CTR—control; VPA—valproic acid exposure; OT—oxytocin; RIS—risperidone; VPA + OT—valproic acid and oxytocin; and VPA + RIS—valproic acid and risperidone; letter a marks results of Tukey’s HSD test).

Furthermore, regarding the anxiety-specific behavioural parameters, we found that the time spent in the top area of the tank decreased in the VPA-treated groups, as compared to the CTR group (Figure 2a). OT and RIS, both solely and combined, showed anxiolytic effects in VPA-treated groups (Figure 2). Otherwise, the exposure to OT visibly increased the time spent in the top area (Figure 2a). No significant differences were obtained while comparing some parameters (latency to the first entry in the top area and frequency between the top and button area) relevant for exploratory behaviour when treating the ASD model with OT and RIS (Figure 2b,c).

### 2.2. The Effect of OT on Aggressive Behaviour

**Figure 3:**
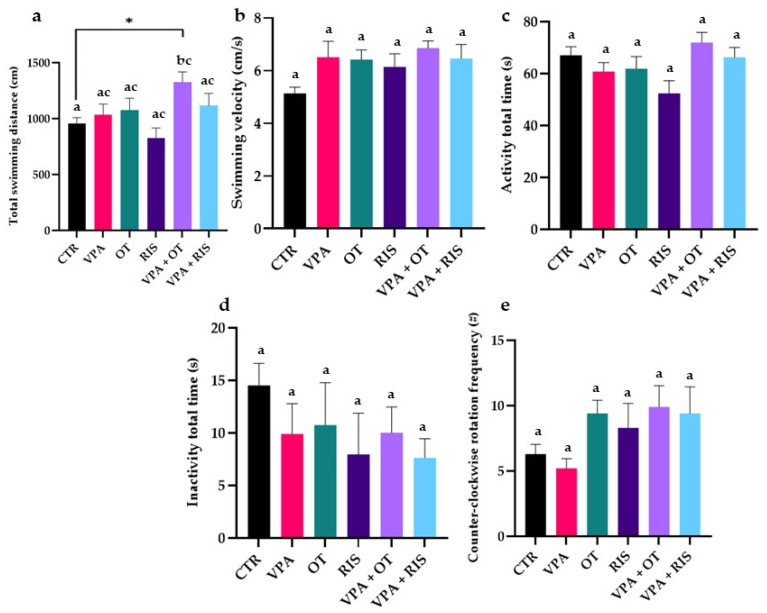
Locomotor performance parameters evaluated in the aggression test: (**a**) Total swimming distance (cm); (**b**) swimming velocity (cm/s); (**c**) activity total time (%); (**d**) inactivity total time (%); and (**e**) counter-clockwise rotation frequency (#). The results are expressed as mean ± SEM (CTR—control; VPA—valproic acid exposure; OT—oxytocin; RIS—risperidone; VPA + OT—valproic acid and oxytocin; and VPA + RIS—valproic acid and risperidone; * *p* < 0.05 in two-way ANOVA test; a–c in Tukey’s HSD test).

In the MBT, we observed that both locomotion and aggression parameters were impaired after VPA treatment. Regarding the locomotory activity, visible increases in general locomotion parameters were observed for all VPA-treated groups, and significantly for the VPA + OT group (*p* < 0.05 in two-way ANOVA test), as compared to the CTR group (Figure 3a,b). Similar effects of both OT and RIS-treated groups were observed during evaluating the activity/inactivity total time, along with the counter-clockwise rotation frequency (Figure 3c–e).

**Figure 4:**
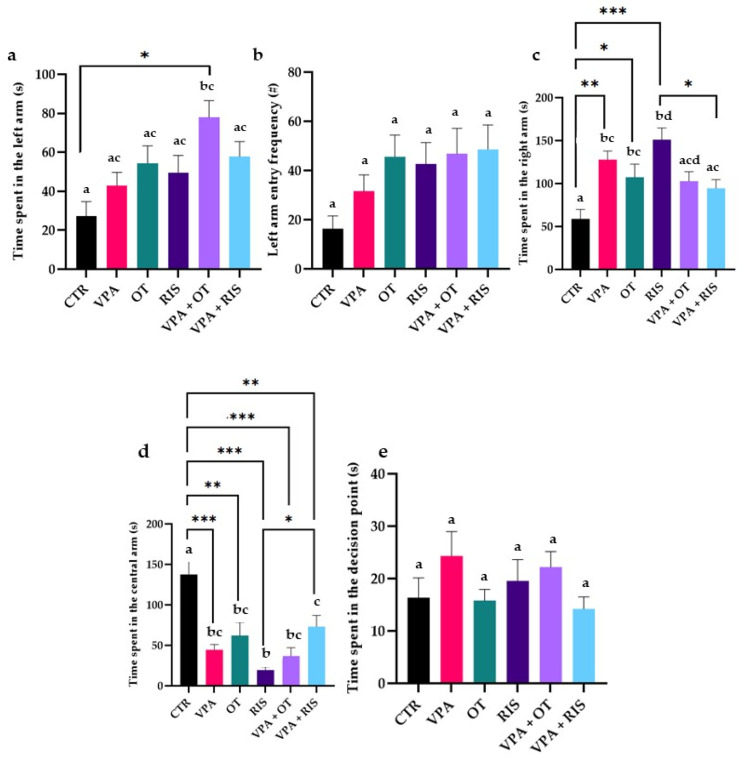
Aggression behavioural parameters evaluated using MBT: (**a**) Time spent in the left arm (s); (**b**) left arm entry frequency (#); (**c**) time spent in the right arm (s); (**d**) time spent in the central arm (s); and (**e**) time spent in the decision point (s). The results are expressed as mean ± SEM (CTR—control; VPA—valproic acid; OT—oxytocin; RIS—risperidone; VPA + OT—valproic acid and oxytocin; and VPA + RIS—valproic acid and risperidone; * *p* < 0.05, ** *p* < 0.01, *** *p* < 0.001 in two-way ANOVA test; a–d in Tukey’s HSD test).

We also observed that the time spent and entry frequency in the arm containing the mirror visibly increased after VPA treatment, as compared to untreated zebrafish. Also, both OT and RIS showed potential in improving aggressive behaviour in VPA-treated zebrafish (Figure 4a,b).

Significant increases in the left arm, as well as in the right arm, central arm, and entry frequency were seen in all experimental groups, as compared to the CTR group (left arm: VPA + OT versus CTR, *p* < 0.01 in two-way ANOVA test; right arm: CTR versus VPA, *p* < 0.01; CTR versus OT, *p* < 0.05; CTR versus RIS, *p* < 0.001; RIS versus VPA + RIS, *p* < 0.05 in two-way ANOVA test; central arm: CTR versus VPA, *p* < 0.001; CTR versus OT, *p* < 0.01; CTR versus RIS, CTR versus VPA + OT, *p* < 0.001; CTR versus VPA + RIS, *p* < 0.01 in two-way ANOVA test). Also, we observed that OT and RIS treatments significantly alleviated anxiety-like behaviour in both untreated and VPA-treated animals, as compared to the CTR group (VPA vs. CTR, *p* < 0.01; OT vs. CTR, *p* < 0.01; RIS vs. CTR, *p* < 0.001, in two-way ANOVA test) (Figure 4c–e).

## 3. Discussion

Since only two atypical antipsychotics are recommended as standard clinical treatments for aggressive behaviour, the current directions of pharmacotherapy in ASD are limited [10,20]. Thus, recent research proposed improving behavioural outcomes and reducing side effects by complementing existing therapeutic strategies [4,21,22]. While RIS and aripiprazole are effective in reducing aggressive behaviour, irritability, and agitation [23], emerging evidence described the superior potential of OT to improve socio-affective impairments in ASD patients, with fewer side effects [24,25,26]. In this context, our study aimed to evaluate the potential of OT, by relation to RIS, to modulate aggressive and anxiety-like behaviours, in a VPA zebrafish model. Consequently, our results showed that OT and RIS had comparable potential to modulate the frequency of aggressive and anxiety-like behaviours and to improve exploratory behaviour, in both healthy and VPA-treated zebrafish.

Our ASD model consisting in VPA treatment for 7 consecutive days manifested in locomotor impairments and increased frequency of anxiety-like behaviour. The neurotoxic effects of VPA involves multiple nervous system pathways, including the monoaminergic system, leading to behavioural deficits, such as socio-affective impairments [27,28,29,30,31]. Moreover, zebrafish studies showed that VPA neurotoxicity could affect zebrafish brains during all developmental stages in a dose and time-dependent manner [32,33,34]. Nevertheless, the correlation between VPA neurotoxicity and aggressive behaviour was previously documented by multiple studies [32,33,35,36]. Strungaru et al. [37] suggested that even spending more time in the proximity of the mirror (i.e., the left arm) could be a sign of potential engagement, and thus a more intense swimming behaviour near the mirror could be associated with a higher level of aggression. In this context, our group has previously reported that a treatment as light as 2.5 mg/L (18 µM) VPA for 2–11 days could result in increased time spent near the mirror, in adult zebrafish [35]. In the current experimental design, we observed that 28.8 mg/L VPA for 7 days led to increased time spent in the arm containing the mirror. Consistent with that, Li et al., 2024 [32] reported both increased time spent near the mirror and mirror attack frequency in adult zebrafish following acute high doses of VPA (500 µM VPA, meaning 144 mg VPA/L, for 4 consecutive days).

Moreover, when OT is administered together with VPA, a possible synergic effect could be observed. In the MBT test, an escalation in aggressive behaviour frequency was observed in the VPA+OT group, as compared to VPA and CTR groups. This apparent paradoxical effect was previously observed by our group when OT was administered to healthy fish [38]. The mechanism through which this synergic effect is obtained could be based on the interaction between cortisol and OT, as previous studies suggested that VPA could modulate anxiety by decreasing cortisol levels [36,39,40]. In parallel, high OT levels in low cortisol conditions could result in increased aggressive behaviour [41]. Moreover, previous rodent studies explained the implications of OT in modulating primary social behaviour as well as stress response [42,43,44,45]. OT-related amygdala activity modulation could thus be the result of both endogenous and exogenous neuroendocrine factors shaping the prosocial and antisocial behaviours in stress conditions [41,42,46,47]. In unfamiliar or stressful contexts, OT has been suggested to promote defensive behaviours [41,46,47].

Furthermore, we observed that when treatment with VPA and OT was performed, increased anxiety-like behaviour frequency, alongside hypolocomotory tendencies, was obtained in the NTT test, as compared with VPA and CTR groups. These results could suggest a synergic effect of OT and VPA in our experimental models. Similar trends were obtained by Robea et al. [38] when continuously treating healthy fish with 33.2 ng/mL (0.033 µM) OT for 7 days. However, their results suggested that shorter OT treatments could be beneficial in improving aggressive behaviour, validating the previous observation of de Los Ángeles Cintado et al. [36] that VPA could modulate anxiety by decreasing cortisol levels, and that high levels of OT could potentiate aggressive response [41].

Similar hypolocomotory trends were obtained when RIS was administered solely and in combination with VPA, according to the previously reported sedative effect of RIS. However, some studies reported that RIS at a similar dose was not able to affect locomotory performance, suggesting that environmental conditions could influence the stress response in RIS treatment conditions (novel environment, or social stimuli) [48,49]. On the other hand, Lungu et al. [17] recently reported that a much smaller dose of RIS (0.08 mg/L [17] versus 0.17 mg/L in our study) could lead to hypolocomotion (i.e., reduced swimming velocity), yet not acknowledging a sedative effect. These differences could be due to subtle modulatory mechanisms of the studied pharmacological compounds (VPA, OT, RIS). The mechanisms of action include the modulation of serotonergic and dopaminergic receptors [50,51,52,53,54,55]. Regarding aggressive behaviour, despite very different mechanisms of action, OT and RIS produce similar behavioural responses. While RIS blocked dopaminergic and stimulated serotonergic receptors to improve aggressive behaviour in humans [19], OT and dopamine could directly interact in determining aggressive behaviour [56,57,58,59]. In ASD, as well as in other aggressivity related disorders, increased dopamine levels and decreased serotonin levels lead to sustained aggressive behaviour [19]. In this context, Petersson et al. [59] reported that OT could increase the dopamine release in the amygdala modulating stress response.

In ASD patients, OT could also modulate socio-affective behaviour by increasing cerebral activity in areas associated with stress response, which are rich in OT receptors [60,61,62,63]. The beneficial effect of OT on improving ASD-associated irritability and aggressive behaviour was acknowledged, yet a correlation between anxiety and aggressive behaviour could limit OT action [20,25,64,65,66,67,68]. In this context, Pfundamair et al. [69] reported a significant correlation between improving aggressive behaviour and anxiety levels, i.e., OT-driven aggressive response was obtained in low anxiety, but not in high anxiety individuals. Accordingly, we showed that the VPA anxiolytic effect could prevent OT action against aggressive behaviour in our VPA zebrafish model.

Several limitations of our study need to be mentioned. Firstly, the present study adds value to the understanding of the neurobiology of ASD, yet animal model experiments should be translated into clinical practice with great caution, as the translational significance could be limited by inherent species-specific differences in neurobiology and behaviour (zebrafish versus human). Secondly, our preliminary results only focussed on general observation of aggressive behaviour, as evaluated by MBT. Yet, several studies have reported that zebrafish could exhibit several subtypes of aggressive behaviour, including impulsive and reactive aggressive behaviour, similarly to non-human mammalian species [18]. In this context, future studies should focus on the more subtle behavioural responses, as different aggressive response subtypes may be modulated by VPA, OT, RIS and other treatments in zebrafish models.

## 4. Materials and Methods

### 4.1. Animals and Housing Conditions

Sixty adult wild-type zebrafish (*Danio rerio*, 3–4 months, sex ratio 1:1) were purchased from a local breeder. Previously to any experimental procedures, the fish were accommodated for 2 weeks in a 50 L glass tank. Afterwards, the fish were randomly assigned to 6 groups and left to accommodate for 7 days in the experimental tanks under standard laboratory conditions (water temperature: 27 ± 1 °C, pH: 7.5, dissolved oxygen: 7.20 mg/L, daily replacement) in 14:10 light to dark cycles. Standard fish food formulation (TetraMin flakes, Melle, Germany) was used to feed the fish twice daily, according to the recommended food/weight ratio. Zebrafish maintenance and experimental procedures were performed according to the Recommendation of the European Union Commission of 18 June 2007, and the Directive of the European Parliament and of the Council of the European Union of 22 September 2010. All the procedures were approved by the Local Ethics Committee of the Faculty of Biology of Alexandru Ioan Cuza University of Iasi (no. 122/12 May 2025).

### 4.2. Drugs and Treatments

VPA (Sigma Aldrich, Darmstadt, Germany), OT (10 UI/mL, Pasteur Institute, Filipesti, Romania), and RIS (Sigma Aldrich, Darmstadt, Germany) solutions were prepared extemporaneously at convenient concentrations to ease the administration in the housing tanks (with water volumes at 5 L each). All the treatments were performed concomitantly, for 7 consecutive days. All doses were selected based on available guidelines and previous studies [38,48,70,71,72] and consisted of the following: 28.8 mg/L VPA, 33.2 ng/mL OT, and 170 μg/L RIS. The treatments were administered in the experimental housing tanks, while the water was replaced daily.

### 4.3. Experimental Design

**Figure 5:**
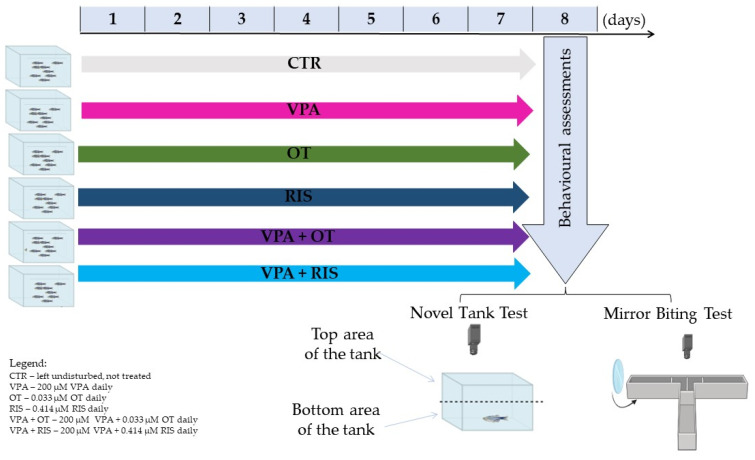
Schematic representation of the experimental design.

Following the accommodation period, fish were randomly assigned to 6 experimental groups (*n* = 10/group): CTR—control; VPA (28.8 mg/L valproic acid); OT (33.2 ng/mL oxytocin); RIS (170 µg/L risperidone); VPA + OT (28.8 mg/L valproic acid and 33.2 ng/mL oxytocin); and VPA + RIS (28.8 mg/L valproic acid and 170 µg/L risperidone). An overview of the experimental design and procedures can be seen in Figure 5.

### 4.4. Behavioural Assessments

At the end of the last day of treatments, standard behavioural tests (novel tank test, NTT and mirror biting test, MBT) were performed to evaluate exploratory, anxiety-like, and aggressive behaviours.

Novel tank test

NTT is commonly used to evaluate locomotory performance and anxiety-like behaviour [73]. A 6 L rectangular tank divided into 2 observational areas (from a side view: bottom and top areas) was used. After 30 s of acclimatisation, the fish freely explored the tank for 4 min. The zebrafish behaviour was video-recorded and processed using EthoVision XT 16 software (Wageningen, The Netherlands). Several behavioural parameters, such as total swimming distance (cm), swimming velocity (cm/s), activity/inactivity total time (s), the frequency of freezing episodes (#), and counter-clockwise rotations (#) were used to describe locomotory performance. On the other hand, the time spent in the top area of the tank (s), the latency to the first entry in the top area (s), and the frequency of top/bottom transition (#) were used to evaluate anxiety-like behaviour.

Mirror biting test

MBT was used to assess the frequency of aggressive behaviour, according to previous experience and guidelines [35,38]. A T-shaped maze consisting of 3 arms (central, left, and right) was used. A mirror was placed at the end of the left arm to simulate the presence of a conspecific. Since animals are not able to recognise their reflection in the mirror, they could respond with aggression to the possible opponent [37,74,75]. After 30 s of accommodation at the starting point, the behaviour of the tested fish was observed for 4 min. The zebrafish activity was recorded and processed using the EthoVision XT 16 software (Wageningen, The Netherlands). The specific behavioural parameters for aggressive behaviour recorded included the time spent near the mirror (s), which indicates the time spent in the proximity of the stimulus and actively interacting with it. In parallel, the possibility of aggressive behaviour tendency was assessed by the left arm entry frequency (#) parameter, which counts the number of individual returns to the left arm. Additionally, the time spent in the other segments (s) (time spent in the right arm (s); time spent in the central arm (s); and time spent in the decision point (s)) measured the time spent avoiding the stimulus, alongside decision-making cognitive processes. In addition, the standard locomotion parameters (total swimming distance (cm), swimming velocity (cm/s), activity/inactivity total time (s), and counter-clockwise rotations (#)) were measured to evaluate general locomotor activity and ensure that the observed behavioural changes were not due to alterations in movement capacity.

### 4.5. Statistical Analysis

The numerical data obtained by analysing the behavioural parameters were statistically processed using the GraphPad PRISM software (version 10.0.0 for Windows, Boston, MA, USA). The results were expressed as mean ± SEM. A two-way ANOVA test, followed by post hoc analyses (Tukey’s HSD test), was performed to test the multiple comparisons between the groups. An F value for which *p* was less than 0.05 was considered statistically significant.

## 5. Conclusions

The pharmacotherapy of neurodevelopmental disorders increasingly emphasises the use of potential modulators of the neuronal pathways controlling socio-affective behaviour. Our results showed that, in a VPA zebrafish model, OT and RIS have the potential to modulate aggressive behaviour. Also, we observed that both OT and RIS improved anxiety-like behaviours in VPA-treated and healthy zebrafish.

## Data Availability

The raw data supporting the conclusions of this article will be made available by the authors on request.
